# Biophysical characterization and crystal structure of the Feline Immunodeficiency Virus p15 matrix protein

**DOI:** 10.1186/1742-4690-10-64

**Published:** 2013-06-24

**Authors:** Jennifer Serrière, Xavier Robert, Magali Perez, Patrice Gouet, Christophe Guillon

**Affiliations:** 1 Laboratoire de Biocristallographie et Biologie Structurale des Cibles Thérapeutiques, IBCP-BMSSI, UMR 5086 CNRS Université de Lyon, SFR BioSciences Gerland-Lyon Sud, 7 Passage du Vercors, 69367 Lyon, Cedex 07, France

**Keywords:** FIV, p15 matrix protein, Gag, DLS, Structure, Interface, Biological dimer

## Abstract

**Background:**

Feline Immunodeficiency Virus (FIV) is a viral pathogen that infects domestic cats and wild felids. During the viral replication cycle, the FIV p15 matrix protein oligomerizes to form a closed matrix that underlies the lipidic envelope of the virion. Because of its crucial role in the early and late stages of viral morphogenesis, especially in viral assembly, FIV p15 is an interesting target in the development of potential new therapeutic strategies.

**Results:**

Our biochemical study of FIV p15 revealed that it forms a stable dimer in solution under acidic conditions and at high concentration, unlike other retroviral matrix proteins. We determined the crystal structure of full-length FIV p15 to 2 Å resolution and observed a helical organization of the protein, typical for retroviral matrix proteins. A hydrophobic pocket that could accommodate a myristoyl group was identified, and the C-terminal end of FIV p15, which is mainly unstructured, was visible in electron density maps. As FIV p15 crystallizes in acidic conditions but with one monomer in the asymmetric unit, we searched for the presence of a biological dimer in the crystal. No biological assembly was detected by the PISA server, but the three most buried crystallographic interfaces have interesting features: the first one displays a highly conserved tryptophan acting as a binding platform, the second one is located along a 2-fold symmetry axis and the third one resembles the dimeric interface of EIAV p15. Because the C-terminal end of p15 is involved in two of these three interfaces, we investigated the structure and assembly of a C-terminal-truncated form of p15 lacking 14 residues. The truncated FIV p15 dimerizes in solution at a lower concentration and crystallizes with two molecules in the asymmetric unit. The EIAV-like dimeric interface is the only one to be retained in the new crystal form.

**Conclusion:**

The dimeric form of FIV p15 in solution and its extended C-terminal end are characteristic among lentiviral matrix proteins. Crystallographic interfaces revealed several interactions that might be involved in FIV replication. Further studies are needed to better understand their biological relevance in the function of FIV Gag during viral replication.

## Background

The Feline Immunodeficiency Virus (FIV) is an enveloped virus from the family *Retroviridae*, genus lentivirus, which also contains the human immunodeficiency virus (HIV), equine infectious anemia virus (EIAV) and simian immunodeficiency virus (SIV). FIV infection induces an immunodeficiency syndrome in infected domestic cats or wild felids, which resembles HIV-induced AIDS. Therefore, this syndrome has been dubbed feline AIDS. FIV is an important veterinary issue as it infects up to 30% of domestic cats in some parts of the world with apparently no evolution of host resistance [1,2]. Moreover, FIV has been described as a useful model for the development of AIDS vaccines, antiretroviral drugs, and non-pathogenic gene therapy vectors [3,4]. However, compared to primate lentiviruses, many fundamental aspects of FIV biology are not well understood, and no structural data are available except for two of the viral enzymes, the dUTP-pyrophosphatase [5], and the protease [6,7].

Matrix protein (MA), p15 in FIV or EIAV and p17 in HIV or SIV, is the N-terminal subunit of the retroviral Gag polyprotein. This Gag polyprotein is involved in the architecture of the viral particle, which is common to HIV and FIV and is essential for infectivity of the virus [8-10]. The MA subunit of Gag is also involved in recruiting the viral envelope glycoproteins onto virions [8-10]. In FIV, HIV or SIV, the N-terminal domain of Gag undergoes a co-translational covalent modification of the MA N-terminus with a myristoyl group [11-15]. This myristoylation, together with a highly basic patch of amino acid residues located at the N-terminus of the MA, directs the Gag polyprotein to the plasma membrane (PM), mediating the association between Gag and the inner leaflet of the PM lipid bilayer, where Gag forms a hexagonal network [16]. The N-terminal end of Gag for some retroviruses, including EIAV, is not myristoylated, and some N-terminal ends bear a different chemical modification [17,18]. Interestingly, FIV appears to be an exception among nonprimate lentiviruses because of its capacity to be myristoylated [19].

After the budding of virus particles from host cells, the Gag polyprotein is cleaved by the viral protease into its different subunits during maturation [20]. In the mature particle, the capsid and the nucleocapsid subunits of the Gag polyprotein make a compact RNA-containing conically shaped capsid inside the virion, in contrast to the MA protein, which remains at the inner surface of the virus membrane.

In the HIV model, the N-terminal myristoyl group of MA can adopt sequestered and exposed conformations [21] consistent with a myristoyl switch mechanism for regulating membrane binding [22-25]. The formation of MA trimers in HIV can trigger the myristoyl switch [21]. While HIV MA proteins associate in solution and in crystal structures as trimers, they organize onto membranes in a hexamer arrangement [26]. These observations have led to the suggestion that the MA trimers incorporate into Gag hexamers, with the trimers forming nodes that interconnect the hexamers [27].

Previous studies have determined the three-dimensional structure of HIV p17, SIV p17 and EIAV p15 by nuclear magnetic resonance spectroscopy (NMR) [21,28-30] and X-ray crystallography [13,31,32]. MA proteins appear to be globular proteins with a compact fold made of five α-helices. In addition, the HIV protein contains a highly basic platform, which allows the targeting of Gag polyproteins to the plasma membrane [14,15]. These structural studies have also demonstrated that p17 HIV and p17 SIV can form trimers [13,32]. However, some retroviral matrix proteins, such as EIAV MA, are dimer units in crystal structures [31,33]. In fact, these conformational differences between the lentiviral proteins may be correlated with their mode of association. Hatanaka *et al.* suggested that the dimeric conformation observed for EIAV p15 could correspond to a weakened membrane-binding state and proposed a new alternative membrane-binding mechanism [31]. The comparison of the atomic structure of FIV p15 with those of matrix proteins of other retroviruses, such as HIV or EIAV, will help to define the specificity of the molecular mechanisms of FIV assembly.

To characterize the FIV p15 structure, we over-expressed FIV p15. This allowed us to perform the first studies on the oligomeric state of the FIV p15 protein and to determine the crystal structure of full-length FIV p15. The structure of a C-terminal-truncated FIV p15 was solved in turn to assess the role of this region in the oligomerization process. In conclusion, we discuss the conformational differences observed between FIV p15 and the matrix protein of other lentivirus. In addition, we also discuss the possible role of the flexible C-terminus of FIV p15 in the replication cycle.

## Results

### Biochemical and biophysical characterization of the FIV p15 oligomeric state

**Figure 1:**
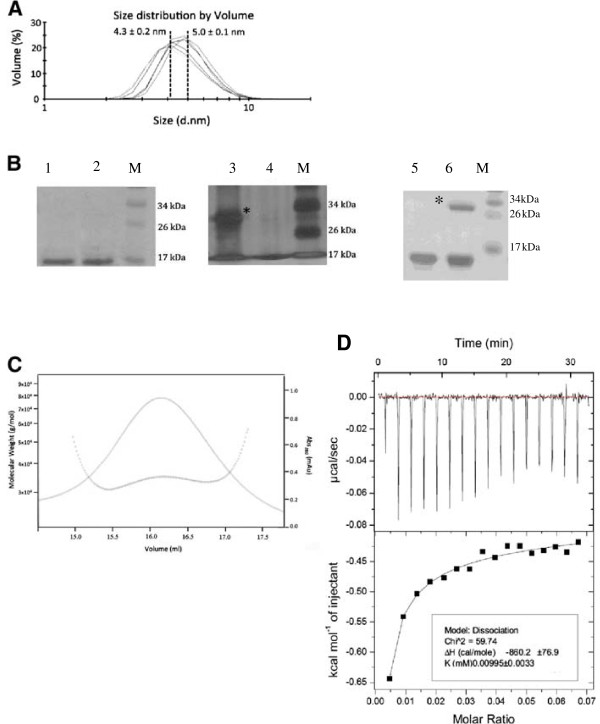
**Analysis of the oligomeric state of the p15-His protein. (A)** DLS spectrogram obtained with the p15-His protein at 3 and 6 mg/ml in 50 mM sodium phosphate pH 7.4 buffer (left curves) and 50 mM MES pH 6 buffer (right curves), respectively. **(B)** Chemical cross-linking with BS^3^ of p15 with or without 6-His tag. Lanes 1 to 4: p15-His at-3.3 and 3 mg/ml (lane 1 and 2) or 6 and 6.5 mg/ml (lane 3 and 4), in 50 mM MES pH 6 buffer (lane 1 and 3) and 50 mM sodium phosphate pH 7.4 buffer (lane 2 and 4). Lanes 5 and 6: p15 without 6-His tag at 6 mg/ml in 50 mM sodium phosphate pH 7.4 buffer (lane 5) or 50 mM MES pH 6 buffer (lane 6). M is the molecular weight marker. The asterisk indicates the expected size for the dimeric form. **(C)** The SEC-MALLS spectra of the molecular weight (dotted line) and absorbance at 280 nm (plain lines) versus elution volume. The p15 protein in 50 mM MES pH 6 buffer and at 6 mg/ml was loaded into the column. **(D)** Dissociation constant of the p15-His dimer measured by ITC. The thermogram (top panel) and the plotted titration curve (bottom panel) were obtained with a Microcal ITC200. The solid line (bottom panel) represents the fitting of the data by the built-in dimer dissociation model.

Retroviral matrix proteins oligomerize into dimers, trimers or hexamers [21,26,31]. Thus, we evaluated the oligomerization properties of FIV p15. The FIV p15 protein was analyzed by DLS (dynamic light scattering) to determine its oligomerization state in solution at different concentrations (3 mg/ml and 6 mg/ml) and pH values (pH 6 and pH 7.4) (Figure 1A). A single peak was observed in each experiment, which confirmed the homogeneity of the protein solution. At 6 mg/ml, the average diameter of the FIV p15 protein in 50 mM sodium phosphate pH 7.4 was 4.3 nm with a standard deviation of 0.2 nm (Figure 1A). This value is consistent with a monomeric form of FIV p15 [34]. In contrast, the protein in 50 mM MES pH 6 presented an average diameter of 5 nm and a standard deviation of 0.1 nm (Figure 1A), suggesting that the protein was mainly in its dimeric form at 6 mg/ml in this more acidic buffer. This was confirmed by cross-linking experiments on the same samples using bis(sulfosuccinimidyl)suberate (BS^3^). These experiments demonstrated that the FIV p15 protein in 50 mM sodium phosphate pH 7.4 was mainly monomeric, whereas a dimeric state was observed for the protein in 50 mM MES pH 6 (Figure 1B). These results are in a good agreement with our experimental data obtained by DLS. It is noteworthy that the protein was monomeric in both buffers at a lower concentration (3 mg/ml in 50 mM sodium phosphate pH 7.4 or 3.3 mg/ml in 50 mM MES pH 6, Figure 1B). Thus, the oligomeric state of FIV p15 protein depends both on the buffer and the protein concentration, shifting from a monomeric to a dimeric state when the acidity of the buffer and the protein concentration increase. The presence of the histidine tag was not responsible for the formation of the dimeric form, as the protein without the tag was also dimeric at 6 mg/ml in MES buffer (Figure 1B).

We confirmed this oligomeric behavior of p15 in solution by submitting a sample of his-tagged protein at 6 mg/ml in 50 mM MES pH 6 to SEC-MALLS (size-exclusion chromatography with multi-angle laser light scattering) (Figure 1C). The FIV p15 protein was eluted as a single peak from the gel-filtration column, demonstrating a single oligomeric state of the protein. According to the refracting index and the light scattering measurements, this oligomer had a MW value approximately 36,000 Da (Figure 1C), confirming the presence of dimers. These data demonstrated that FIV p15 is in a dimeric conformation in solution under mild acidic conditions and at a high protein concentration. In these conditions, the dissociation constant (Kd) of this dimer was measured using isothermal titration calorimetry (ITC) as being around 10 μM (Figure 1D).

### Structure determination of the FIV full-length p15 protein by X-ray crystallography

FIV full-length p15 protein at 6 mg/ml in MES buffer pH 6 crystallizes in the presence of sodium acetate pH 4.6 and PEG in the space group P2_1_2_1_2 with one molecule in the asymmetric unit. Synchrotron data were collected to 2 Å resolution. The phase problem was solved by molecular replacement using SIV p17 (PDB ID: 1ECW) as the search model [32]. The FIV p15 structure was refined to a final R_work_ of 19% (R_free_ = 25.5%) (Table 1).

**Table 1:** Data collection and refinement statistics

***Data collection***	**FIV p15**	**FIV p15-Δ120**
Beamline	Proxima 1 (SOLEIL, Paris)	ID-29 (ESRF, Grenoble)
Detector	Pilatus 6 M	Pilatus 6 M
Wavelength (Å)	0.98011	0.97239
Data collection temperature (K)	100	100
Space group	P2_1_2_1_2	P2_1_
Unit cell parameters	a = 53.0 Å, b = 71.2 Å, c = 28.2 Å	a = 28.5 Å, b = 71.3 Å, c = 57.1 Å
	α = β = γ = 90°	α = γ = 90°, β = 99.6°
Matthews coefficient /% solvent	1.89 / 34.90%	1.95 / 37.05%
Resolution range (Å)	20-2.0 (2.05-2.0)	20-2.7 (2.77-2.7)
Total number of reflections	52,897	20,503
Number of unique reflections	7,647 (543)	6,139 (448)
Rsym (%)	9.2 (45.4)	8.1 (37.6)
<I>/σ < I>	16.32 (4.78)	16.9 (4.80)
Completeness (%)	99.8 (99.8)	98.1 (97.8)
Redundancy	6.9	3.3
***Refinement***	**FIV p15**	**FIV p15-Δ120**
Resolution range (Å)	19.31-2.0 (2.29-2.0)	19.68-2.7 (3.40-2.7)
Number of unique reflections	7,647 (2,357)	6,138 (1,571)
Rwork (%)	19.05 (21.11)	21.51 (23.48)
Rfree (%)	24.60 (25.72)	27.87 (30.02)
Number of protein atoms	1,032	1,808
Number of water/ethylene glycol molecules/PEG molecules	28/4/0	24/0/2
Mean B-factor (Å^2^)	19.40	27.65
Coordinate deviations		
RMSD bond lengths (Å)	0.006	0.002
RMSD angles (°)	0.930	0.417
Ramachandran plot		
Favorable (%)	96.88	96.15
Allowed (%)	3.12	3.85
Disallowed (%)	0.00	0.00
PDB ID	4IC9	4ICA

Outermost shell values are indicated in parentheses.

**Figure 2:**
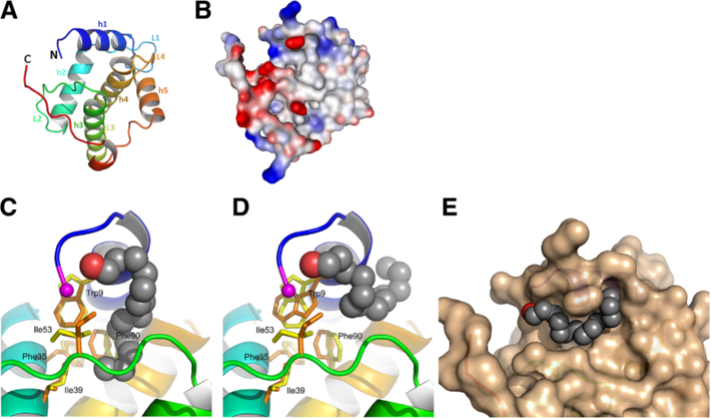
**Overview of the FIV p15 protein. (A)** Structure of FIV p15, with the secondary structure elements colored blue-to-red from the N- to the C-terminus and helices numbered h1 to h5. Loops l1 to l4 between the helices are represented as well as the N- and C- termini. **(B)** Surface representation of the electrostatic properties of FIV p15. The electrostatic potential is shown with positive charges in blue and negative charges in red. The arrow indicates the putative myristoyl-binding pocket of FIV p15. **(C)** and **(D)** Docking prediction of the myristoyl insertion in FIV p15 in **(C)** and out **(D)** the hydrophobic pocket. The myristoyl group is displayed as a van der Waals representation with gray spheres and its carboxyl functional group in red. The N-terminal glycine residue is represented in magenta. The residues involved in the interaction with the myristoyl group are labeled and displayed in yellow or orange at their initial positions and their positions after the docking experiment, respectively. The backbone of the protein is colored as in **(A)**. **(E)** The representation of the surface of FIV p15 after docking of the myristoyl group in the external groove. Myristoyl is displayed as in **(C)** and (**D)**.

The structure consists of five α-helices (numbered h1 to h5 following the HIV convention), which give rise to the close packing of adjacent helices typical for retroviral matrix proteins (Figure 2A). The three amino-terminal residues (Gly4-Gln5-Gly6) and the twenty carboxy-terminal residues (Met113 to Gln133) are mainly unstructured and extended (Figure 2A). Despite its intrinsic flexibility, the C-terminal end (Met113 to Gln133) is clearly observed in the electron density map because of the stabilizing crystal contacts of the C-terminal residues 130 to 132 with the complementary monomer.

An analysis of the electrostatic potential of FIV full-length p15 revealed a highly basic platform, with helix h1 having amphipathic character (residues Arg7 to Cys16) (Figure 2B). Numerous arginine and lysine residues within the 30 N-terminal amino acids form a highly basic region that provides a motif presumably to interact with the acidic phospholipid head groups, as described for HIV p17 [21,35]. This creates a strong dipole across the molecule and a positively charged surface for membrane interaction (Figure 2B). As with HIV, a hydrophobic cavity formed by the side chains of helices h1 to h4 is observed in the FIV p15 protein (Figure 2B). Our docking studies demonstrated that a myristoyl group can be placed in this cavity with its carboxyl end in the vicinity of the N-terminus of p15, providing the local adjustment of residues from helices h1, h2 and h4 (Trp9, Phe35, Ile39, Ile53, and Phe90, Figure 2C). These residues are located at the same positions as the residues interacting with the N-terminal myristoyl of the HIV MA (Ser9, Ile34, Ser38, Leu51 and Leu85, respectively [21]). The estimated binding free energy of the myristoyl group is similar for FIV and HIV (-2.9 kcal/mol and -3.7 kcal/mol, respectively). One striking feature, when the myristoyl group is inserted in the hydrophobic pocket of FIV p15, is the predicted motion of Trp9 and Ile53, which rotate from their initial position (Figure 2C). Of interest, our docking experiments revealed a second putative binding site for the myristoyl group that wraps around the first turn of helix h1 (Figure 2D) in a groove located at the surface of the protein (Figure 2E). This location involves the interaction of the myristoyl group with residues Trp9, Ala12, Arg15, Glu55 and Leu95 of helix h1 and loops l2 and l4.

**Figure 3:**
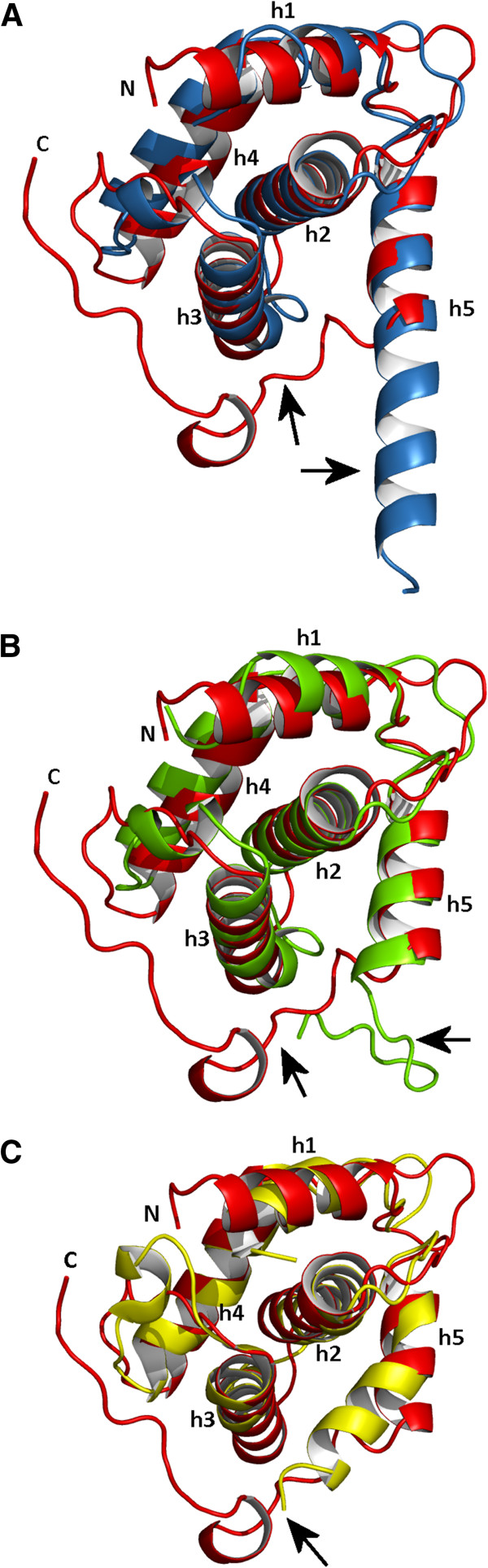
**Structural comparison of four lentiviral matrix protein.** The superimposition of the matrix proteins of FIV (red) with **(A)** HIV (PDB ID: 1HIW, blue), **(B)** SIV (PDB ID: 1ECW, green) and **(C)** EIAV (PDB ID: 1HEK, yellow) matrix proteins. The N- and C-termini are indicated, and the helices are numbered h1 to h5 as in Figure 2A. Black arrows highlight the structural differences at the C-terminal end of the matrix proteins.

**Figure 4:**
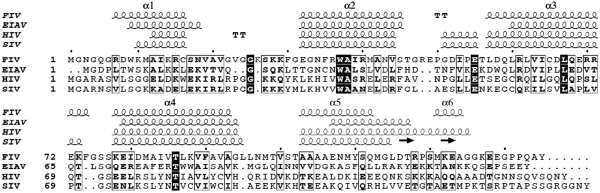
**Structure-based sequence alignment of four lentiviral matrix proteins.** Alignment between FIV p15, EIAV p15, HIV p17 and SIV p17. The conserved residues are boxed and the physico-chemical equivalent residues are on a light gray background. Helices (squiggles along the sequence) of the crystal structures are shown for FIV p15 (this paper), EIAV p15 (PDB ID: 1HEK), HIV p17 (PDB ID: 1HIW) and SIV p17 (PDB ID: 1ECW). The alignment was performed with ESPript web server [67].

FIV p15, despite having a low sequence similarity with HIV, EIAV and SIV MA (18%, 17% and 15% sequence identities, respectively) is strikingly similar in structure to these lentiviral matrix proteins and can be superposed to each with a root mean square deviation (RMSD) of 1.9 Å, 2.5 Å, and 2.1 Å between Cα pairs, respectively (Table 2). However, the superimposition of the lentiviral MA structure reveals a major difference around the C-terminal end of MA (Figure 3). The C-terminal end of FIV p15 is mainly unstructured and extended (Figures 2 and 3), whereas a long helix h5 is observed in HIV p17 (Figure 3A), and a short β-hairpin is reported in SIV MA (Figure 3B). The C-terminal end of EIAV p15 is not visible in the electron density maps and is absent from its structure (Figure 3C). Another difference is the presence of longer loops in FIV as compared with EIAV, HIV and SIV because of insertions in the FIV sequence (Figure 4). As these loops are important to the stabilization of HIV and SIV trimers, the longer loops may be related to the differences observed in the FIV p15 oligomerization.

**Table 2:** Structural comparisons with other lentiviral matrix proteins

**Protein**	**PDB ID**	**RMSD**^**1**^	**N**_**align**_^**2**^	**%seq**^**3**^
EIAV MA	1HEK	2.5 Å	99	17
HIV MA	1HIW	1.9 Å	101	18
SIV MA	1ECW	2.1 Å	105	15

^1^ Root Mean Square Deviation calculated between Cα-atoms of matched residues.

^2^ Number of aligned residues after structure superimposition.

^3^ Sequence identity.

There are 6 conserved residues between the proteins (Figure 4, black squares), most of which are within the helical sections of the molecule, and they may be responsible for maintaining the characteristic matrix architecture. Finally, we can observe in the structure of FIV p15 a cluster of Phe residues (Phe30, Phe35 and Phe90) that form a π-stacking interaction between helices h2 and h4 (data not shown). This hydrophobic interaction is not found in other retroviral matrix proteins and may be implicated in the stabilization of conformation of the FIV p15 protein [36].

### Oligomeric interfaces of FIV p15 in the crystal

Because FIV p15 forms a dimer in solution at acidic pH, and FIV p15 crystals were obtained in an acidic crystallization condition, we performed a search for a biological assembly in the crystal with the PISA server [37]. No dimeric (or higher) assembly was detected. Therefore, we more closely examined the top three dimeric interfaces in terms of buried surface.

**Figure 5:**
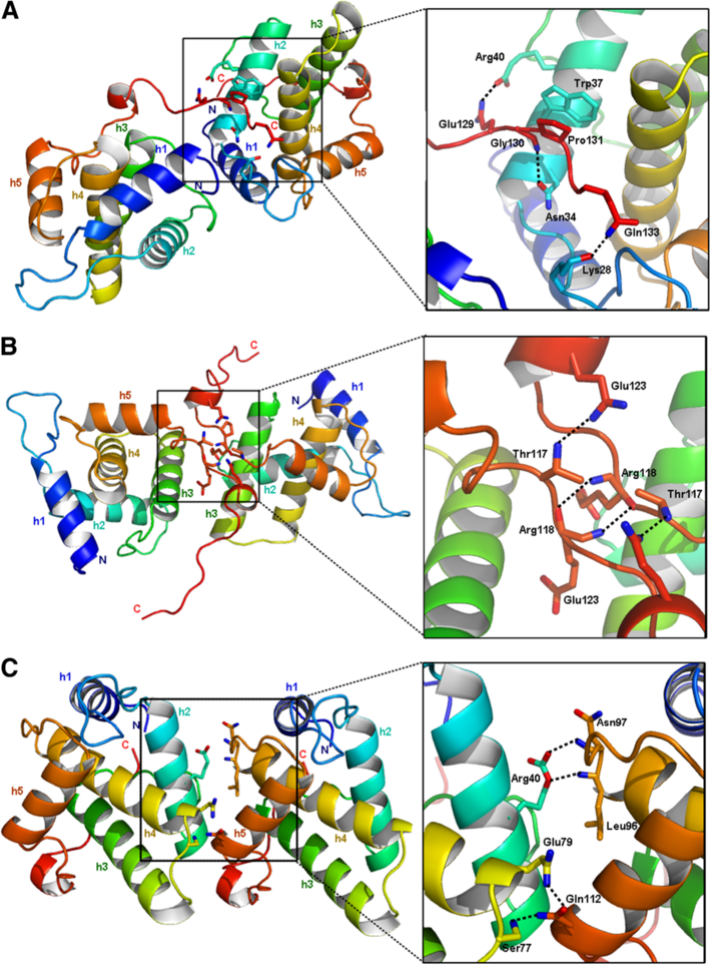
**Crystal-packing interfaces of FIV p15 generated by PISA. (A)** Interface 1 implicating the Trp37 residue in an interaction with the C-terminal end of p15. **(B)** Interface 2 implicating C-terminal antiparallel regions. **(C)** Interface 3 implicating residues from h2, h4, h5 and l3. N- and C-termini are indicated, and helices are numbered h1 to h5 with a coloring scheme identical to Figure 2A. The hydrogen bonds are displayed as dashed lines.

The first interface (interface 1) implicates the conserved residue Trp37, localized at the center of helix h2 and the C-terminal end of a contacting p15 monomer (Gly130 to Pro132) (Figure 5A). The average buried surface area is 543 Å^2^ per monomer. Of interest, the indol group of Trp37 makes extensive contacts with the Pro131 of the complementary molecule *via* hydrophobic stacking. This maintains the C-terminal end of FIV p15 in an extended conformation in the crystal, which is stabilized by three hydrogen bonds (Figure 5A and Table 3).

**Table 3:** Intermolecular contacts in the crystallographic interface for full-length FIV p15

**Interface**	**Buried area per monomer**	**Interacting residues and atoms (first monomer)**	**Interacting residues and atoms (second monomer)**	**Distance (Å)**
Interface 1	543 Å^2^	Arg40-NH2	Glu129-OE1	2.2
		Lys28-NZ	Gln133-OE1	2.3
		Asn34-NH2	Gly130-O	2.9
Interface 2	497 Å^2^	Thr117-OG1	Glu123-OE2	2.8
		Arg118-N	Arg118-O	3.1
		Arg118-O	Arg118-N	3.1
		Glu123-OE2	Thr117-OG1	2.8
Interface 3	401 Å^2^	Leu96-O	Arg40-NE	2.7
		Asn97-O	Arg40-NH2	3.2
		Gln112-OE1	Ser77-OG	2.3
		Gln112-NE2	Glu79-OE2	2.6

The second interface (interface 2) implicates an antiparallel assembly made up by residues of the C-terminal end via a 2-fold symmetry axis (Figure 5B). Its average buried surface area is 497 Å^2^ per monomer. The assembly structure has bonds between three residues (Thr117-Arg118-Glu123) and their 2-fold symmetry related counterparts (Figure 5B and Table 3).

The third interface (interface 3) involves an interaction between loop l3 and helix h5 from one monomer with loop l3 and helix h4 from the other monomer (Figure 5C and Table 3). This interface displays an estimated buried surface area of 401 Å^2^ per monomer. The interface 3 resembles the interface of EIAV dimers [31] (Additional file 1: Figures S2A & S2B). Moreover, it is the only interface with a favorable P-value calculated by PISA [37] for the observed solvation free energy gain (p = 0.287) compared to interface 1 (p = 0.487) or interface 2 (p = 0.603).

**Figure 6:**
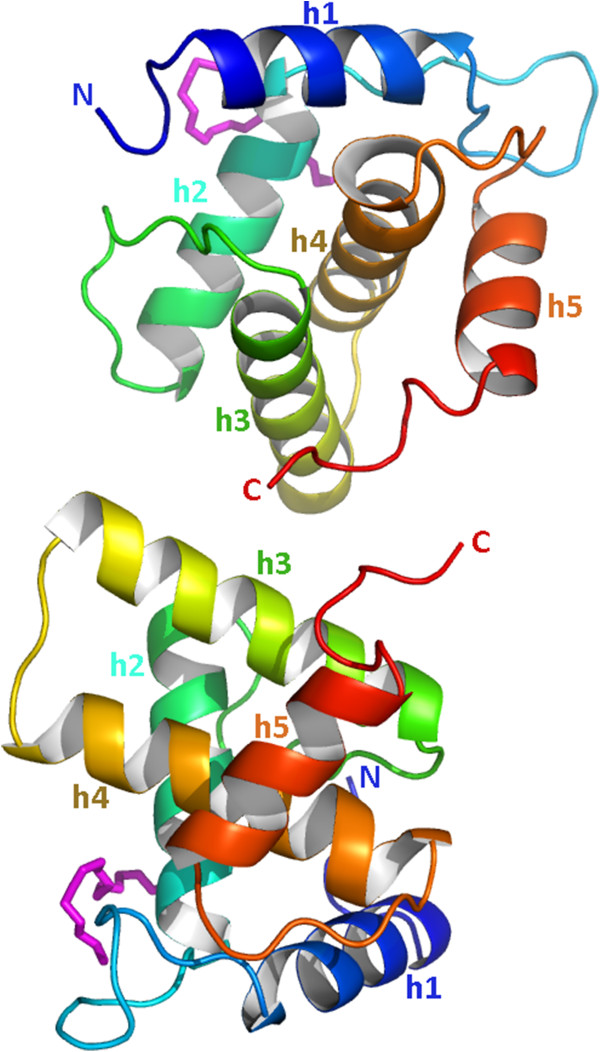
**Crystal structure of p15-Δ120.** The color scheme of the two p15 subunits is identical to Figure 2A. N- and C-termini are indicated, and the helices are numbered h1 to h5. The two PEG molecules are displayed in magenta.

**Figure 7:**
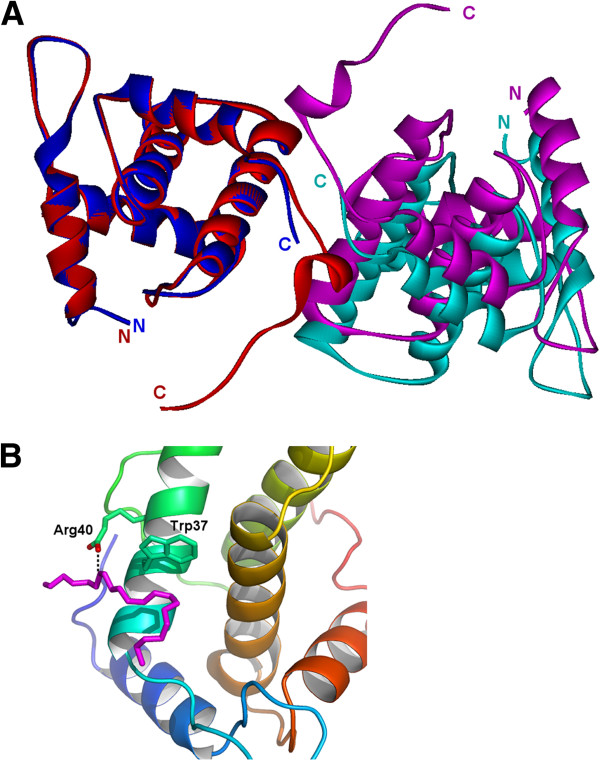
**Comparison of FIV p15 structures. (A)** Superimposition of the structure of p15-Δ120 (chain A in blue, chain B in cyan) with interface 3 in the full-length p15 crystal (chain A in red, symmetric molecule in magenta). The superimposition was performed on the Cα of both chains A using COOT. N- and C- termini are indicated for each chain. **(B)** The close-up view of the interaction of the PEG molecule (magenta) with the Trp37 and Arg40 residues of p15-Δ120.

To investigate which interface could be the biological dimer that we observe *in vitro*, we constructed a shortened form of FIV p15, p15-Δ120, which is truncated after Ser120 to remove the residues involved in interface 1. The p15-Δ120 truncated protein was stable and was still able to generate dimers in solution in MES buffer, even at a lower protein concentration (3 mg/ml, Additional file 1: Figure S1A). Crystals of p15-Δ120 could be grown, and data could be collected to 2.6 Å resolution. The crystal space group for p15-Δ120 is different (P2_1_) from the full-length protein (P2_1_2_1_2), and 2 monomers are present in the asymmetric unit (Figure 6). The truncation of the last 14 residues does not alter the overall conformation of the protein, as demonstrated by the RMSD of 0.5 Å between the Cα pairs in the truncated protein and the full-length protein. The assembly in the asymmetric unit of p15-Δ120 resembled the interface 2 observed with the full-length protein. However, this interaction does not involve the main chain of the Arg118 of both molecules, as with the full-length protein, but instead involves the side chain of residues Arg118 of chain A with the side chain of residue Glu79 of chain B of p15-Δ120. This results in a shift between the two chains, with a displacement of centroid distances of approximately 6 Å and a rotation of approximately 11° of the chain B of p15-Δ120 compared to the symmetric chain of the full-length protein in interface 2 (Figure 7A). As expected, interface 1 is no longer present in the crystal packing of p15-Δ120 as the C-terminal residues involved in this interface with the full-length protein were truncated. However, Trp37, which was involved in the hydrophobic interaction with Pro131 in interface 1 (Figure 5A), is not free in the p15-Δ120 crystal. Instead, this conserved residue (Figure 4) demonstrates hydrophobic interactions with a PEG fragment from the crystallization solution in both monomers of the asymmetric unit (Figure 7B). This interaction, stabilized by hydrogen bonds between Arg40 and the PEG chain, buries an area of 232 Å^2^. The presence of this PEG interaction with Trp37 in place of the C-terminal end of full-length FIV p15 suggests that this residue could represent a platform for hydrophobic interactions with FIV p15.

Finally, the interface 3 observed in the full-length protein is maintained in the crystal packing of p15-Δ120 with a similar buried surface and intermolecular interactions as the full-length protein (Additional file 1: Figure S2C).

**Figure 8:**
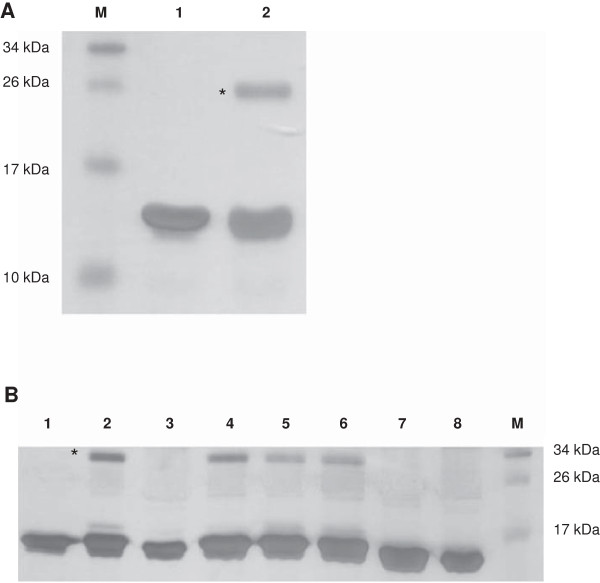
**Investigation of p15 dimer formation. (A)** Chemical cross-linking with BS^3^ of p15-Δ120-R40S at 6 mg/ml in 50 mM MES pH 6 buffer in the absence (lane 1) or presence (lane 2) of BS^3^. **(B)** Chemical cross-linking of p15 with BS^3^ in the presence of MES (lane 2), crystallization condition 25 of PEG suite (0.2 M sodium acetate pH 4.6, 20% w:v PEG 3350, lane 3), sodium acetate 0.2 M pH 4.6 (lane 4), and 2.5%, 5%, 10% and 20% w:v PEG 3350 (lanes 5 to 8 respectively). Lane 1 is the negative control of p15 without BS^3^.

In order to investigate the crystal assembly, we constructed a mutated form of the truncated p15 protein with the long basic Arg40 residue, which is observed in the crystallographic interfaces 1 and 3, mutated to a short polar serine residue to remove the terminal interacting NH2 of Arg40(p15-Δ120-R40S). Cross-link experiments showed that this mutant was still able to generate dimers in solution in MES buffer (Figure 8A). Because none of the crystallographic interfaces could be linked to the known properties of retroviral matrix proteins and to the biochemical data obtained with our constructs *in vitro*, we wondered whether the dilution of the protein in the crystallization condition could have altered the dimeric interface. The presence of a high concentration of PEG in the solution did not allow us to investigate the presence of a dimer using MALLS, DLS or ITC experiments because of the viscosity of the sample. However, cross-linking experiments demonstrated that the dimer of full-length p15, although stable in MES pH 6, dissociates when mixed to the crystallization condition to become a monomer (Figure 8B). This dissociation of the dimer was not due to an interference of the crystallization condition with the cross-linking agent, as control dimeric proteins were still cross-linked in the crystallization condition of p15 (Additional file 1: Figure S1B). We therefore investigated which component of the crystallization condition (0.2 M sodium acetate pH 4.6, 20% w:v PEG 3350) was responsible for this dissociation. Incubation of p15 with 0.2 M sodium acetate pH4.6 alone did not dissociate the dimer, excluding a role of the pH on p15 dimer dissociation (Figure 8B). On the opposite, the presence of high concentrations of PEG (>10% w:v, Figure 8B) was associated with the disappearance of the dimeric form. It has to be noted that the behavior of p15-Δ120 in its own crystallization condition was not tested because it contains Tris that interferes with BS^3^ cross-linking.

## Discussion

### FIV p15 dimerization is pH and concentration dependent: influence on myristoyl group exposure

To our knowledge, this study is the first to characterize the oligomeric forms of FIV p15, using a combination of dynamic light scattering (DLS), chemical cross-linking, and SEC-MALLS experiments. Our results demonstrate for the first time that the oligomeric state of FIV p15 is dependent both on protein concentration and on the buffer used. The dimerization of FIV p15 protein at high concentration and under acidic conditions is different from the reported trimerization of HIV p17 [13].

Here, we provide evidence that the oligomeric state of the non-myristoylated FIV p15 protein is modulated by pH. The monomeric protein shifts to a dimeric form when the pH decreases. This dimer was stable as demonstrated by its dissociation constant around 10 μM as measured by ITC. These results support the hypothesis that deprotonation, by increasing the pH to 7.4, inhibits the formation of oligomers for FIV p15, as previously observed in the case of myristoylated HIV p17 [38]. Intracellular pH often fluctuates (from 6.3 to 7.8) in response to cell growth, development and apoptosis [39-41] and varies in sub-cellular compartments. In particular, a significant decrease in pH from 7.2 to as low as 6.0 has been observed in cells infected by HIV [42]. This acidification could initiate the multimerization of Gag at the end stage of the viral cycle to lead to viral particle assembly. At early stage of replication (i.e. after virus entry in a cell with a more neutral pH), deprotonation would lead to a shift towards a monomeric form of p15 during the release of the viral core in the infected cell. Therefore, the matrix protein could act as a “pH sensor” implicated in a pH-dependent assembly and disassembly of retroviral particles. Such a role of pH variations in Gag oligomerization remains to be confirmed *in cellulo*.

The oligomeric state of the FIV p15 protein is also concentration-dependent *in vitro*. In the case of HIV, it is thought that the Gag polyprotein is primarily monomeric at low concentrations and is therefore largely cytoplasmic because of the sequestration of the myristoyl group [43]. As its concentration increases, a greater fraction of Gag is driven into multimeric forms, resulting in myristoyl exposure and efficient membrane binding. The virus might exploit this mechanism to confine Gag polyproteins in specific cellular locations [38]. However, the myristoyl switch mechanism is significantly different for the matrix proteins of HIV-1, HIV-2 and EIAV [31,44], indicating that key differences in the myristoyl switch mechanism may exist [38]. This mechanism could also be different for FIV. According to docking experiments, the insertion of the myristoyl group in the hydrophobic pocket of FIV p15 is possible, with similar binding free energy compared to the estimation based on the structure of HIV myristoylated p17, and involves residues at the same positions. However, the sequestration of the myristoyl group in FIV p15 is associated with the motion of the side chain of Trp9, which is involved, in turn, in an aromatic stacking against of the Phe35 and Phe90 side chains. These aromatic residues are absent in HIV but well conserved between circulating FIV strains, with Phe90 sometimes substituted with another aromatic residue (Tyr), suggesting the importance of this aromatic stacking for the function of p15. More experiments should be performed to evaluate if the sequestration of the myristoyl for FIV p15 involves an aromatic switch, which would represent specificity for FIV p15 compared to HIV p17.

Another feature revealed by the docking experiments is the possibility for the myristoyl group to bind in a groove at the surface of FIV p15. This groove involves residues Asp8, Ala12, Glu55, and Leu95, which are highly conserved between FIV strains. Moreover, this groove is also present in the structure of the HIV MA protein [21]. It is noteworthy that the residues Leu8, Glu12, Glu52 and His89 of HIV p17, located at the same position as those of FIV p15 predicted to interact with the myristoyl group, are present in the groove of HIV p17 and conserved among HIV strains [45]. Although an NMR study of myristoylated HIV p17 did not reveal this conformation of the myristoyl group [21], the presence of this groove in both structures, involving conserved residues in both viruses, could reflect a common role for this groove in accommodating the myristoyl group across lentiviral species; for example during the decapsidation process when the matrix protein has to leave the inner leaflet of the plasma membrane to participate in the formation of the preintegration complex.

### Structural specificities of the C-terminus of FIV p15

We have determined the crystal structure of the FIV p15 full-length protein and of a form that lacks its C-terminal end (p15-Δ120). Both structures are similar, with a RMSD of 0.5 Å between Cα pairs. Like the MA proteins of other lentivirus, the FIV p15 protein has five α-helices. However, these helices have different lengths compared to the published structures of the lentiviral MA proteins for HIV [13], SIV [32], and EIAV [31]. The structure of the full-length FIV p15 allowed us to observe in the electron density maps the complete C-terminus of the protein up to residue 134, and only residues 2 and 3 of the N-terminus were missing for the full-length protein. However, these N-terminal residues 2 and 3 were visible in the structure of the truncated form p15-Δ120.

As stated above, we were able to observe the complete, extended C-terminal end of the full-length FIV p15 due to crystal contacts between Pro131 and Trp37 of the complementary monomer. One cannot exclude a possible influence of the C-terminal histidine tag on the flexibility that we observe for the C-terminal region of p15. However, this seems unlikely for two reasons. First, the protein without the 6-His tag displayed the same biochemical properties in solution than with the tag, as demonstrated by cross-link experiments. Second, the presence of the His-tag does not modify the structure of the C-terminal region in the truncated form p15-Δ120 compared to the full-length protein. The structure of the C-terminal end appears to be an important feature for retroviral matrices as it differs depending on the virus. Indeed, the C-terminal region following helix h5 is extended in the FIV p15 structure, which is in line with what has been demonstrated for EIAV [31], where the flexible C-terminus is not observed in the electron density map. In contrast, a structured C-terminus with a short β-hairpin is observed in SIV MA [32] and with a long helix h5 in HIV MA [13] (Figure 3). However, the structures of HIV MA, as determined by NMR (PDB ID: 1HMX and 1TAM), indicate that this region is disordered [30,46]. Thus, the extended conformation of the FIV p15 C-terminus could represent a biological feature of the protein, and the well-ordered structures of the HIV and SIV p17 C-terminus could represent crystallization artifacts [47]. This is underlined by the fact that the C-terminal portion of MA is not compactly folded in Gag virus-like particles [48,49]. In such particles, it is projected into the external environment and used for the connection between MA and CA in the Gag polyprotein. Although it is not essential for the self-assembly process of Gag precursor molecules [50], this extended C-terminal region of MA could be necessary to grant accessibility to the viral protease that cleaves Gag between MA and CA during the maturation of the virus. Finally, it has been shown that the C-terminal end of HIV MA may be implicated in the early steps of the viral life cycle [51]. Therefore, the flexibility of this end may be involved in the destabilization of viral particles after virus entry into the cell.

### Crystal contacts reveal potential interactions platforms in FIV p15

Unlike SIV, HIV and EIAV MA, FIV p15 appears monomeric in the crystal, although it is dimeric in the protein solution used for crystallization. We could demonstrate that this monomerization of p15 during crystallization was linked to the dissociation of the dimer in the presence of PEG in the crystallization condition. Therefore, it is not clear how the crystallographic contacts we observe might participate to the biological dimer. For example, we demonstrated a shift from a monomer to a dimer in MES buffer at pH 6 but not in phosphate buffer at pH 7.4. This suggests charged residues in the interface of the biological dimer in solution. Unfortunately, the three interfaces that we observed in the crystal involved charged residues that are conserved among FIV strains. It is therefore not possible to discriminate between these interfaces based on their conservation between FIV strains or differences in their protonation susceptibility.

However, several conclusions can be drawn from the analysis of the three FIV p15 interfaces that were identified in the crystals. In interface 1, Trp37 is implicated in the interaction with C-terminal Pro131 of the complementary monomer (Figure 5A). This Trp residue is highly conserved among lentiviral species and has been shown to be implicated in the dimer interface of EIAV MA [31]. For HIV and SIV MA, this residue is accessible to the surface of the protein, allowing potential interactions with biological partners. This might also apply to FIV p15 where Trp37 is exposed and participates in contacts with a symmetric protein in the full-length FIV p15 protein crystal (Figure 5A). When deleting the C-terminal region that interacts with Trp37 in the p15-Δ120 construct, the deleted C-terminus is replaced in the crystal of the truncated p15-Δ120 by a PEG molecule, which interacts with this tryptophan (Figure 7B), suggesting that this residue represents a strong hydrophobic interacting platform for FIV p15. This binding of a fragment of PEG to this region might also provide the basis of our observation that PEG dissociates p15 dimers *in vitro.* This suggests that this hydrophobic platform around Trp37 could be involved in the formation of the p15 dimer, which would be destabilized by a PEG molecule that would compete with a p15 monomer for binding to this platform. Previous studies have shown that this Trp contributes to the PI(4,5)P2 binding site of HIV MA [44]. Moreover, the mutation of this residue in the case of the HIV totally abolishes the assembly of viral particles, suggesting structural implications for this residue in the matrix protein [52]. Hydrophobic Trp-Pro interactions have also been described in protein/protein interactions during HIV replication, for example in the interaction of HIV CA with cyclophilin A [53,54]. Thus, in the case of FIV p15, Trp37 could be a platform for p15 dimerization, binding of PI(4,5)P2 and/or binding of other proteins such as those involved in the pre-integration complex (PIC). Although the mechanism whereby the MA protein is recruited into the PIC and contributes to nuclear import remains unclear, it has been shown that the C-terminal residues of HIV MA can interact with the integrase protein [55]. The influence of the FIV p15 C-terminal domain in the formation of the PIC during FIV replication should therefore be investigated. However, although interface 1 suggests the importance of Trp37 for the function of the FIV p15 protein, the residue is not involved in the biological dimeric interface of p15 as demonstrated by the detection of matrix dimers for the truncated p15-Δ120 protein that lacks the Pro131 interacting with Trp37.

In interface 2, an antiparallel interface, involving residues Thr117, Arg118 and Pro119 and their symmetry-related counterparts, is observed at the center of the dimer interface for the full-length protein but is less tight for the truncated p15-Δ120. It should be noted that SIV also displays an antiparallel interface at its C-terminal end, but this interface is intramolecular, leading to the formation of a C-terminal β-hairpin. It would be interesting to investigate the role of such an antiparallel interaction in the function of retroviral matrix proteins.

A third dimeric interface was identified in FIV p15 crystals, both in the full-length protein and the truncated p15-Δ120. At first glance, interface 3 resembles the interface found in the EIAV dimer [31], with the orientation of the two N-terminal regions in the same direction and the involvement of helices h2 and h5 (Additional file 1: Figure S2). However, it displays several differences. Indeed, EIAV presents a dimeric interface along a 2-fold axis, with a slight rotation between the two monomers resulting in curved crystal packing [31], but interface 3 of FIV p15 results from a translation of one monomer to the next monomer (Figure 5C). However, this is the only interface of FIV p15 that i) is observed in both full-length and p15-Δ120 crystals, ii) is not modified by the C-terminal truncation of FIV p15, and iii) orients the N-terminus (and therefore the myristoyl group) and the basic patch of helix h1 towards the same orientation as the two monomers of the dimer. According to the model of HIV assembly, this concerted orientation is the only one that would allow the simultaneous interaction of both monomers with the internal surface of the plasma membrane and permit the radial arrangement of Gag polyprotein during virus assembly. Interestingly, the buried surface area of this interface (401 Å^2^) is similar to the buried surface area (410 Å^2^) of the protein interface leading to the formation of the SIV trimers [32].

In the absence of native dimers in the crystal structures, due to the dissociation of p15 dimers by the PEG present in the crystallization condition, a specific and systematic mutagenesis study will need to be performed to identify the residues involved in the formation of the dimers. The elucidation of the biological dimeric interface of FIV p15 is important, as dimers of FIV p15 proteins could represent intermediates in the biological multimerization of Gag polyproteins into hexamers. Previous data indicates that although HIV MA proteins form 3D crystals as trimers [13], no matrix trimers were visible by electron microscopy analysis and fitting a trimer unit into the hexameric projection structure was difficult to achieve, suggesting that matrix hexamers rather than trimers are the fundamental unit of HIV particles [26]. Three MA dimers could fit in the hexameric model, suggesting that the dimeric form could represent a potential biological state of the matrix protein. An electron microscopy analysis of p15 rearrangements in FIV immature particles and the replacement of the FIV p15 structure in the 3D reconstruction could highlight the molecular contacts involved in the formation of the viral matrix and confirm this model.

## Conclusion

In summary, the dimeric form of FIV p15 in solution and its extended C-terminal end in the crystal structure are original features of this protein when compared to HIV, SIV and EIAV. Although the dimeric interface remains to be identified, the presence of a Trp residue as an interacting platform is likely important for the function of FIV p15. Further study of these characteristics will be needed to better understand their biological relevance in the context of FIV replication and their peculiarity among lentiviral matrix proteins.

## Methods

### Plasmid constructs

The FIV p15 open reading frame of the Petaluma strain (GenBank accession number: M25381.1) was amplified from 0.1 μg of the plasmid FIV-34TF10 obtained through the NIH AIDS Research and Reference Reagent Program.

For the PCR amplification of the full-length protein, we used the forward primer (5'-CATATGGGGAATGGACAGGGGCGAGATTGG -3', *NdeI* site underlined), and the reverse primer (5’-AGATCTTCAATGATGATGATGATGATGCTCGAGTGAACCTCTTGG-3') using the Phusion PCR kit (Finnzyme) according to the manufacturer’s protocol, with 25 cycles of PCR and a hybridization temperature of 50°C. A truncated form of the p15 protein (p15-Δ120) was obtained using the same procedure, with reverse primer 5’-GCTCGAGTGAACCTCTTGGAACCAGAGATGGCCTAGTGTCTAATC-3’. A second PCR round was performed on all constructs with the same forward primer and with reverse primer 5'-AGATCTTCAATGATGATGATGATGATGCTCGAGTGAACCTCTTGG-3' introducing the *Bgl2* site (underlined) as well as a C-terminal 6-His Tag. The PCR products were digested with *NdeI* and *Bgl2* and inserted into the MCS of the pRSET-B prokaryotic expression vector (Invitrogen) digested with the same enzymes. This creates the pRSET-p15 expression plasmid for our p15 constructs that encode proteins with a 6-His tag at the C-terminal end of the protein. The plasmids were transformed into *E. coli* BL21(DE3)pLysS cells (Lucigen) for protein expression.

### Expression of His-tagged p15

Cells transformed with pRSET-p15 were grown at 37°C in Hyper Broth (HB) medium (AthenaES™) supplemented with 25 μg/ml of Ampicillin. Transformed cells were grown up in 10 ml of HB for 20 h at 37°C with agitation. This over-grown culture was then added to 1 L of HB supplemented with 25 μg/ml of Ampicillin in a 5 L baffled flask. Cell growth was monitored by optical density (OD) measurements at 600 nm. When culture reached an OD_600_ of 0.6, expression of FIV p15 proteins was induced by the addition of isopropyl-β-D-thiogalactopyranoside (IPTG, Euromedex) to a final concentration of 2 mM. After 20 h of induction at 25°C, cells were collected by centrifugation at 10,000 × g for 8 min, and the wet pellet was stored at -20°C overnight.

### Purification of His-tagged p15

Two steps of purification were performed to purify FIV p15 proteins: a nickel affinity followed by a size exclusion chromatography.

A total amount of 10 g of frozen cell pellet from 1 L culture was resuspended in LEW buffer (Lysis-Equilibration-Wash, 50 mM NaH_2_PO_4_, 300 mM NaCl), pH 8, with lysozyme (Sigma) to a final concentration of 1 mg/ml, anti-protease cocktail 1X (Halt™ Protease Inhibitor Cocktail, Thermo Scientific) and DNase I (Sigma) at 2U/ml final, for 1 h on ice. Then, the resuspended pellet was pushed 3 times through a high-pressure chamber, using a microfluidizer M-110P (Microfluidics) at 17,000 psi. The bacterial lysate was clarified by centrifugation at 10,000 × g for 20 min, and the supernatant was filtered through a 0.45 μm membrane. The supernatant was loaded into gravity column packed with 2 g of Ni^2+^-TED resin (Macherey-Nagel) and eluted with LEW buffer pH 8 containing 50 mM of Imidazole, after 3 washes with LEW buffer pH 8. For gel filtration chromatography, the samples from the affinity chromatography containing the p15 protein loaded on a Vivaspin-PES-5 K filter (Sartorius) and centrifuged at 7,000 × g at 4°C until it reached the volume of 10 ml. The gel filtration experiment was then performed in a FPLC system (Äkta purifier, GE Healthcare), using a Superdex 75 TM Hiload 26/60 column (GE Healthcare) and a buffer containing 50 mM sodium phosphate pH 7.4 or 50 mM MES pH 6, in one injection of 10 ml.

His-tagged p15 protein purification fractions were quantified by measuring the absorbance at 280 nm, using a Nanodrop. The identity and the purity of the protein were assessed by SDS-PAGE analysis. Protein was concentrated using a Vivaspin-PES-5 K filter (Sartorius) and centrifuged at 7,000 × g at 4°C until the desired concentration was reached.

### Removal of the 6-His-tag

Purified protein at 1 mg/ml in phosphate buffer was digested overnight at 19°C with 10U of thrombin (Sigma) per mg of protein. After proteolysis, the reaction mix was loaded twice on a Ni-NTA centrifugation column (Proteus) according to the manufacturer’s protocol and the flow-through were collected and pooled. Buffer exchange and protein concentration was then performed as described above for the His-tagged protein.

### Dynamic Light Scattering (DLS)

Purified protein in 50 mM phosphate buffer at pH 7.4 or 50 mM MES buffer at pH 6 was concentrated to 4 and 6 mg/ml respectively by using a Vivapsin-PES-5 K centrifugal concentrator (Sartorius). The DLS experiment was realized on a dynamic light scattering Zetasizer Nano-S ZEN1600 instrument (Malvern Instruments) at 20°C. The samples were previously filtered through a 0.02 μm pore size filter (Nalgene). Each measure is the mean of 12 runs and was repeated three times.

### Chemical cross-linking

Purified p15 proteins at high concentration (6 mg/ml in 50 mM phosphate buffer at pH 7.4 or 6 mg/ml in 50 mM MES buffer at pH 6) and at lower concentration (3 mg/ml in 50 mM phosphate buffer at pH 7.4 or 3.3 mg/ml in 50 mM MES buffer at pH 6) were mixed with a cross-linking agent, the Bis(SulfoSuccinimidyl) Suberate (BS^3^, Pierce). Reaction mixtures containing 7 μg of proteins were incubated with 1 μl of 5 mM BS^3^ (corresponding to a 20:1 (w:w) BS^3^:p15 ratio) for 30 to 45 minutes at room temperature. The reaction was stopped by addition of 1.5 μl of 250 mM Tris-HCl pH 7.5, 30 mM Glycine for 15 minutes at room temperature, and adjusted to a final volume of 10 μl. Cross-linked proteins were denatured by addition of Laemmli sample buffer and cross-linked proteins were submitted SDS-PAGE analysis. To assess the impact of the crystallization condition, p15 proteins at 6 mg/ml in 50 mM MES buffer pH 6) were mixed to crystallization condition, 0.2 M sodium acetate, or increasing concentration of PEG with a ratio 1:1. Control was performed by mixing the protein with MES buffer only with the same ratio 1:1. Murine antibody 11H6H1 [56] and FIV capsid protein p24 [57] were used as control proteins for BS^3^ cross-linking in the crystallization condition.

### SEC-MALLS experiments

To verify the oligomeric state of the protein in solution, FIV p15 was analyzed using a SEC-MALLS (size-exclusion chromatography with multi-angle laser light scattering). We loaded 0.2 ml samples of p15 protein at 6 mg/ml in 50 mM MES pH 6, onto a Superdex 200 10/30 gel-filtration column equilibrated at 0.5 ml/min with 50 mM MES pH 6. The eluate was passed successively through a MiniDawn TREOS-angle light scattering detector (Wyatt) coupled to an Optilab T-rEX refractive index monitor (Wyatt). The data were processed, and molecular masses were calculated using the Astra V software (Wyatt).

### Isothermal Titration Calorimetry (ITC) and estimation of the K_d_

Dilution experiments were conducted as described previously [57] to investigate the dissociation process of the dimer-to-monomer transition using an ITC200 calorimeter (GE Healthcare). Briefly, protein samples of FIV p15 at 6 mg/ml (375 μM) into 50 mM MES pH 6 were used for sequential injections of concentrated protein solution (injection #1: 1.5 μl and injections #2-16: 2.5 μl), spaced at 120 seconds intervals, into the calorimetric cell (200 μl), which initially contained buffer alone (50 mM MES pH 6). Measurements were performed at 25°C, and data were analyzed using a dissociation model in the Origin software according to the manufacturer's instructions (GE Healthcare). The first data point was excluded in the analysis. The binding parameters Δ*H* (reaction enthalpy change in cal/mol) and *K*_*d*_ (dissociation constant in mM) were allowed to float during the fit.

### Crystallization of the FIV p15 protein and data collection

Crystallization conditions were searched using the sitting-drop vapour-diffusion method and commercial kits from Hampton Research, Molecular Dimensions Limited (MDL) and Qiagen. Crystals of FIV p15 were obtained by equilibrating drops of protein (concentrated at 7 mg/ml in 50 mM MES buffer pH 6) mixed 1:1 with the condition 25 of the Qiagen PEGs Suite, consisting of 0.1 M sodium acetate pH 4.6 and 25% (w:v) polyethylene glycol (PEG) 3,000 at 16°C. Tiny rod-shaped crystals with maximum dimension 60 × 30 × 10 μm^3^ were obtained within fifteen days. Crystals were transferred to a cryoprotective solution containing the mother liquor and 10% (v:v) ethylene glycol for 30 sec and plunged into liquid nitrogen prior to data collection. First grown crystals diffracted at best to 3 Å resolution at the SLS beamline PXIII (Villigen PSI, Switzerland) and at the ESRF beamline ID23-2 (Grenoble, France) under cryo-conditions (100 K). Microseeding was necessary to optimize crystals and the best dataset was collected at 100 K to 2 Å resolution at the SOLEIL beamline Proxima 1 (Paris, France). A new crystallization condition was determined for the p15-Δ120 construct. Crystals were obtained in 0.1 M Tris pH 8, 25% (w:v) PEG 6000 (condition 45 of the Qiagen PEGs suite). Crystals were harvested as for the full-length protein and data were collected at 100 K to 2.7 Å resolution at the ESRF beamline ID29.

### Structure determination of FIV p15 and refinement

Diffraction intensities were processed with the programs XDS [58] and XSCALE. The structure of full-length p15 was determined by the molecular replacement method using the program MrBUMP [59] of the CCP4 program suite and the structure of SIV matrix protein (PDB ID: 1ECW) as the search model. Final automated building was performed with ARP/wARP [60], which built 95% of the residues present in the final model. Crystallographic refinement was performed with PHENIX [61] and residues 119-129 were built manually using COOT [62]. The final model is made up by one copy of FIV p15 (chains A, residues 4-133), 4 molecules of ethylene glycol and 28 water molecules. The model shows a good geometry with 2 glycine residues as Ramachandran outliers. Figures were generated with the PyMOL software from Schrödinger. The electrostatic potential map and the molecular surfaces were calculated using DelPhi [63]. Structure of p15 truncated mutant p15-Δ120 was determined using the full-length p15 structure as a lead. The 2 N-terminal residues of p15 could be manually reconstructed using COOT. Two PEG molecules were identified and positioned in the electron density maps using PHENIX and the 7PE ligand coordinates from the PDB as a template. The final model is made up by two copies of p15-Δ120 (chains A and B, residues 2 to 120), 2 molecules of PEG (chains P and Q) and 24 water molecules. This structure shows a good geometry with no Ramachandran outlier. Statistics of the X-ray data are provided in Table 1.

### Docking studies

Molecular docking of the myristoyl group (as ligand) against p15 crystal structure (the receptor) was done using molecular docking program AutoDock 4.2.3 [64,65]. Both molecules were prepared with AutoDockTools 1.5.4 [65] by adding all essential hydrogen atoms and assigning Gasteiger charges. All the 12 active torsions of the ligand were taken into account for the subsequent calculations. Finally, 6 amino acid residues were defined as flexible for the receptor, namely Trp9, Cys16, Phe35, Ile39, Ile53 and Phe90. A grid box of dimensions of 20 × 20 × 20 Å^3^ (with a grid spacing of 0.159 Å), large enough to encompass the pocket, was calculated using the AutoGrid program. Lamarckian genetic search algorithm was employed and a maximum of twenty-five million energy evaluations was performed. After docking completion, solutions were ranked and sorted according to their docked energy using the built-in clustering function. Detailed inspection of the ligand-receptor interactions was carried out with AutoDockTools.

### Alignment of sequence of retroviral matrix proteins

The sequences alignment between retroviral matrix proteins was carried out using the web tool ESPript/ENDscript [66,67]. The best structural homologues are the EIAV p15 protein (PDB ID: 1HEK), the HIV p17 protein (PDB ID: 1HIW) and the SIV p17 protein (PDB ID: 1ECW) with 21%, 18% and 11% of sequence identity with the FIV p15 protein.

## Availability of supporting data

The coordinates and the structure factors are deposited in the Protein Data Bank [68] under the accession numbers 4IC9 and 4ICA for the full-length FIV p15 and p15-Δ120, respectively.

## Competing interests

The authors declare no competing interests.

## Authors’ contribution

JS, PG and CG designed the study. JS, MP, and CG performed the cloning, expression, biophysical characterization and crystallogenesis experiments for both FIV p15 constructs. JS, XR, PG and CG collected, analyzed and interpreted the X-ray diffraction data. XR performed the docking studies. All authors wrote the paper. All authors read and approved the final manuscript.

## Supplementary Material

Additional file 1: Figure S1 **(A)** Cross-linking experiment on p15-Δ120 in MES buffer pH 6 at 3 mg/ml (lane 1 & 4) or 6 mg/ml (lane 2 & 5) in the absence (lanes 1–2) or the presence (lanes 4–5) of cross-linking agent BS^3^. Lane 3: molecular weight marker. **(B)** Chemical cross-linking with BS^3^ of control protein FIV p24 [57] in its own buffer (lane 1) or crystallization condition of p15 (0.2 M sodium acetate pH 4.6, 20% w:v PEG 3350, lane 2). Lane 3: molecular weight marker. The asterisk indicates the expected size for the dimeric forms for each protein. **Figure S2:** Comparison of the dimeric interface of EIAV **(A)** with the interface 3 of full-length p15 **(B)** and p15-Δ120 **(C)**. The color scheme is identical to Figure 2A. Click here for file

## References

[B1] Troyer JL Pecon-Slattery J Roelke ME Johnson W VandeWoude S Vazquez-Salat N Brown M Frank L Woodroffe R Winterbach C Seroprevalence and genomic divergence of circulating strains of feline immunodeficiency virus among Felidae and Hyaenidae species J Virol 2005 79 8282 8294 10.1128/JVI.79.13.8282-8294.2005 15956574 PMC1143723

[B2] Winkler IG Lochelt M Flower RL Epidemiology of feline foamy virus and feline immunodeficiency virus infections in domestic and feral cats: a seroepidemiological study J Clin Microbiol 1999 37 2848 2851 10449463 10.1128/jcm.37.9.2848-2851.1999 PMC85393

[B3] Burkhard MJ Dean GA Transmission and immunopathogenesis of FIV in cats as a model for HIV Curr HIV Res 2003 1 15 29 10.2174/1570162033352101 15043209

[B4] Elder JH Lin YC Fink E Grant CK Feline immunodeficiency virus (FIV) as a model for study of lentivirus infections: parallels with HIV Curr HIV Res 2010 8 73 80 10.2174/157016210790416389 20210782 PMC2853889

[B5] Prasad GS Stura EA McRee DE Laco GS Hasselkus-Light CS Elder JH Stout CD Crystal structure of dUTP pyrophosphatase from feline immunodeficiency virus Protein Sci 1996 5 2429 2437 10.1002/pro.5560051205 8976551 PMC2143329

[B6] Laco GS Schalk-Hihi C Lubkowski J Morris G Zdanov A Olson A Elder JH Wlodawer A Gutschina A Crystal structures of the inactive D30N mutant of feline immunodeficiency virus protease complexed with a substrate and an inhibitor Biochemistry 1997 36 10696 10708 10.1021/bi9707436 9271500

[B7] Wlodawer A Gutschina A Reshetnikova L Lubkowski J Zdanov A Hui KY Angleton EL Farmerie WG Goodenow MM Bhatt D Structure of an inhibitor complex of the proteinase from feline immunodeficiency virus Nat Struct Biol 1995 2 480 488 10.1038/nsb0695-480 7664111

[B8] Freed EO HIV-1 gag proteins: diverse functions in the virus life cycle Virology 1998 251 1 15 10.1006/viro.1998.9398 9813197

[B9] Freed EO Martin MA The role of human immunodeficiency virus type 1 envelope glycoproteins in virus infection J Biol Chem 1995 270 23883 23886 10.1074/jbc.270.41.23883 7592573

[B10] Freed EO Martin MA Domains of the human immunodeficiency virus type 1 matrix and gp41 cytoplasmic tail required for envelope incorporation into virions J Virol 1996 70 341 351 8523546 10.1128/jvi.70.1.341-351.1996 PMC189823

[B11] Dalton AK Ako-Adjei D Murray PS Murray D Vogt VM Electrostatic interactions drive membrane association of the human immunodeficiency virus type 1 Gag MA domain J Virol 2007 81 6434 6445 10.1128/JVI.02757-06 17392361 PMC1900125

[B12] Freed EO Orenstein JM Buckler-White AJ Martin MA Single amino acid changes in the human immunodeficiency virus type 1 matrix protein block virus particle production J Virol 1994 68 5311 5320 8035531 10.1128/jvi.68.8.5311-5320.1994 PMC236481

[B13] Hill CP Worthylake D Bancroft DP Christensen AM Sundquist WI Crystal structures of the trimeric human immunodeficiency virus type 1 matrix protein: implications for membrane association and assembly Proc Natl Acad Sci USA 1996 93 3099 3104 10.1073/pnas.93.7.3099 8610175 PMC39768

[B14] Yuan X Yu X Lee TH Essex M Mutations in the N-terminal region of human immunodeficiency virus type 1 matrix protein block intracellular transport of the Gag precursor J Virol 1993 67 6387 6394 8411340 10.1128/jvi.67.11.6387-6394.1993 PMC238073

[B15] Zhou W Parent LJ Wills JW Resh MD Identification of a membrane-binding domain within the amino-terminal region of human immunodeficiency virus type 1 Gag protein which interacts with acidic phospholipids J Virol 1994 68 2556 2569 8139035 10.1128/jvi.68.4.2556-2569.1994 PMC236733

[B16] Nermut MV Hockley DJ Jowett JB Jones IM Garreau M Thomas D Fullerene-like organization of HIV gag-protein shell in virus-like particles produced by recombinant baculovirus Virology 1994 198 288 296 10.1006/viro.1994.1032 8259664

[B17] Henderson LE Sowder RC Smythers GW Oroszlan S Chemical and immunological characterizations of equine infectious anemia virus gag-encoded proteins J Virol 1987 61 1116 1124 3029406 10.1128/jvi.61.4.1116-1124.1987 PMC254072

[B18] Schultz AM Henderson LE Oroszlan S Fatty acylation of proteins Annu Rev Cell Biol 1988 4 611 647 10.1146/annurev.cb.04.110188.003143 3058168

[B19] Elder JH Schnolzer M Hasselkus-Light CS Henson M Lerner DA Phillips TR Wagaman PC Kent SB Identification of proteolytic processing sites within the Gag and Pol polyproteins of feline immunodeficiency virus J Virol 1993 67 1869 1876 8383214 10.1128/jvi.67.4.1869-1876.1993 PMC240254

[B20] Coffin JM Retroviridae: the viruses and their replication. 3rd ed edn 1996 Philadelphia, Pa: Lippincott-Raven Publishers

[B21] Tang C Loeliger E Luncsford P Kinde I Beckett D Summers MF Entropic switch regulates myristate exposure in the HIV-1 matrix protein Proc Natl Acad Sci USA 2004 101 517 522 10.1073/pnas.0305665101 14699046 PMC327179

[B22] Zhou W Resh MD Differential membrane binding of the human immunodeficiency virus type 1 matrix protein J Virol 1996 70 8540 8548 8970978 10.1128/jvi.70.12.8540-8548.1996 PMC190946

[B23] Hermida-Matsumoto L Resh MD Human immunodeficiency virus type 1 protease triggers a myristoyl switch that modulates membrane binding of Pr55(gag) and p17MA J Virol 1999 73 1902 1908 9971769 10.1128/jvi.73.3.1902-1908.1999 PMC104431

[B24] Paillart JC Gottlinger HG Opposing effects of human immunodeficiency virus type 1 matrix mutations support a myristyl switch model of gag membrane targeting J Virol 1999 73 2604 2612 10074105 10.1128/jvi.73.4.2604-2612.1999 PMC104015

[B25] Resh MD A myristoyl switch regulates membrane binding of HIV-1 Gag Proc Natl Acad Sci USA 2004 101 417 418 10.1073/pnas.0308043101 14707265 PMC327161

[B26] Alfadhli A Huseby D Kapit E Colman D Barklis E Human immunodeficiency virus type 1 matrix protein assembles on membranes as a hexamer J Virol 2007 81 1472 1478 10.1128/JVI.02122-06 17108052 PMC1797500

[B27] Forster MJ Mulloy B Nermut MV Molecular modelling study of HIV p17gag (MA) protein shell utilising data from electron microscopy and X-ray crystallography J Mol Biol 2000 298 841 857 10.1006/jmbi.2000.3715 10801353

[B28] Saad JS Miller J Tai J Kim A Ghanam RH Summers MF Structural basis for targeting HIV-1 Gag proteins to the plasma membrane for virus assembly Proc Natl Acad Sci USA 2006 103 11364 11369 10.1073/pnas.0602818103 16840558 PMC1544092

[B29] Tang C Ndassa Y Summers MF Structure of the N-terminal 283-residue fragment of the immature HIV-1 Gag polyprotein Nat Struct Biol 2002 9 537 543 12032547 10.1038/nsb806

[B30] Massiah MA Starich MR Paschall C Summers MF Christensen AM Sundquist WI Three-dimensional structure of the human immunodeficiency virus type 1 matrix protein J Mol Biol 1994 244 198 223 10.1006/jmbi.1994.1719 7966331

[B31] Hatanaka H Iourin O Rao Z Fry E Kingsman A Stuart DI Structure of equine infectious anemia virus matrix protein J Virol 2002 76 1876 1883 10.1128/JVI.76.4.1876-1883.2002 11799182 PMC135893

[B32] Rao Z Belyaev AS Fry E Roy P Jones IM Stuart DI Crystal structure of SIV matrix antigen and implications for virus assembly Nature 1995 378 743 747 10.1038/378743a0 7501025

[B33] Riffel N Harlos K Iourin O Rao Z Kingsman A Stuart D Fry E Atomic resolution structure of Moloney murine leukemia virus matrix protein and its relationship to other retroviral matrix proteins Structure 2002 10 1627 1636 10.1016/S0969-2126(02)00896-1 12467570

[B34] Wilkins DK Grimshaw SB Receveur V Dobson CM Jones JA Smith LJ Hydrodynamic radii of native and denatured proteins measured by pulse field gradient NMR techniques Biochemistry 1999 38 16424 16431 10.1021/bi991765q 10600103

[B35] Chukkapalli V Hogue IB Boyko V Hu WS Ono A Interaction between the human immunodeficiency virus type 1 Gag matrix domain and phosphatidylinositol-(4,5)-bisphosphate is essential for efficient gag membrane binding J Virol 2008 82 2405 2417 10.1128/JVI.01614-07 18094158 PMC2258911

[B36] Grimes J Basak AK Roy P Stuart D The crystal structure of bluetongue virus VP7 Nature 1995 373 167 170 10.1038/373167a0 7816101

[B37] Krissinel E Henrick K Inference of macromolecular assemblies from crystalline state J Mol Biol 2007 372 774 797 10.1016/j.jmb.2007.05.022 17681537

[B38] Fledderman EL Fujii K Ghanam RH Waki K Prevelige PE Freed EO Saad JS Myristate exposure in the human immunodeficiency virus type 1 matrix protein is modulated by pH Biochemistry 2011 49 9551 9562 20886905 10.1021/bi101245j PMC3032006

[B39] Schuldiner S Rozengurt E Na+/H + antiport in Swiss 3T3 cells: mitogenic stimulation leads to cytoplasmic alkalinization Proc Natl Acad Sci USA 1982 79 7778 7782 10.1073/pnas.79.24.7778 6961450 PMC347431

[B40] Moolenaar WH Tsien RY van der Saag PT de Laat SW Na+/H + exchange and cytoplasmic pH in the action of growth factors in human fibroblasts Nature 1983 304 645 648 10.1038/304645a0 6410286

[B41] Gottlieb RA Giesing HA Zhu JY Engler RL Babior BM Cell acidification in apoptosis: granulocyte colony-stimulating factor delays programmed cell death in neutrophils by up-regulating the vacuolar H(+)-ATPase Proc Natl Acad Sci USA 1995 92 5965 5968 10.1073/pnas.92.13.5965 7541139 PMC41622

[B42] Makutonina A Voss TG Plymale DR Fermin CD Norris CH Vigh S Garry RF Human immunodeficiency virus infection of T-lymphoblastoid cells reduces intracellular pH J Virol 1996 70 7049 7055 8794349 10.1128/jvi.70.10.7049-7055.1996 PMC190755

[B43] Perez-Caballero D Hatziioannou T Martin-Serrano J Bieniasz PD Human immunodeficiency virus type 1 matrix inhibits and confers cooperativity on gag precursor-membrane interactions J Virol 2004 78 9560 9563 10.1128/JVI.78.17.9560-9563.2004 15308748 PMC506924

[B44] Saad JS Ablan SD Ghanam RH Kim A Andrews K Nagashima K Soheilian F Freed EO Summers MF Structure of the myristylated human immunodeficiency virus type 2 matrix protein and the role of phosphatidylinositol-(4,5)-bisphosphate in membrane targeting J Mol Biol 2008 382 434 447 10.1016/j.jmb.2008.07.027 18657545 PMC2581411

[B45] Kuiken CL, Foley B, Leitner T, Hahn BH, Apetrei C, Mizrachi I, Mullins JI, Rambaut A, Marx PA, Wolinksy S, Korber B HIV Sequence Compendium 2010 2010 Los Alamos: Theoretical Biology and Biophysics, Los Alamos National Laboratory, Los Alamos

[B46] Matthews S Barlow P Clark N Kingsman S Kingsman A Campbell I Refined solution structure of p17, the HIV matrix protein Biochem Soc Trans 1995 23 725 729 8654825 10.1042/bst0230725

[B47] Massiah MA Worthylake D Christensen AM Sundquist WI Hill CP Summers MF Comparison of the NMR and X-ray structures of the HIV-1 matrix protein: evidence for conformational changes during viral assembly Protein Sci 1996 5 2391 2398 10.1002/pro.5560051202 8976548 PMC2143307

[B48] Fuller SD Wilk T Gowen BE Krausslich HG Vogt VM Cryo-electron microscopy reveals ordered domains in the immature HIV-1 particle Curr Biol 1997 7 729 738 10.1016/S0960-9822(06)00331-9 9368755

[B49] Wilk T Gross I Gowen BE Rutten T de Haas F Welker R Krausslich HG Boulanger P Fuller SD Organization of immature human immunodeficiency virus type 1 J Virol 2001 75 759 771 10.1128/JVI.75.2.759-771.2001 11134289 PMC113972

[B50] Chazal N Gay B Carriere C Tournier J Boulanger P Human immunodeficiency virus type 1 MA deletion mutants expressed in baculovirus-infected cells: cis and trans effects on the Gag precursor assembly pathway J Virol 1995 69 365 375 7983731 10.1128/jvi.69.1.365-375.1995 PMC188584

[B51] Yu X Yu QC Lee TH Essex M The C terminus of human immunodeficiency virus type 1 matrix protein is involved in early steps of the virus life cycle J Virol 1992 66 5667 5670 1501299 10.1128/jvi.66.9.5667-5670.1992 PMC289135

[B52] Lee YM Tang XB Cimakasky LM Hildreth JE Yu XF Mutations in the matrix protein of human immunodeficiency virus type 1 inhibit surface expression and virion incorporation of viral envelope glycoproteins in CD4+ T lymphocytes J Virol 1997 71 1443 1452 8995670 10.1128/jvi.71.2.1443-1452.1997 PMC191201

[B53] Gamble TR Vajdos FF Yoo S Worthylake DK Houseweart M Sundquist WI Hill CP Crystal structure of human cyclophilin A bound to the amino-terminal domain of HIV-1 capsid Cell 1996 87 1285 1294 10.1016/S0092-8674(00)81823-1 8980234

[B54] Yoo S Myszka DG Yeh C McMurray M Hill CP Sundquist WI Molecular recognition in the HIV-1 capsid/cyclophilin A complex J Mol Biol 1997 269 780 795 10.1006/jmbi.1997.1051 9223641

[B55] Gallay P Swingler S Song J Bushman F Trono D HIV nuclear import is governed by the phosphotyrosine-mediated binding of matrix to the core domain of integrase Cell 1995 83 569 576 10.1016/0092-8674(95)90097-7 7585960

[B56] Serrière J Dugua J-M Bossus M Verrier B Haser R Gouet P Guillon C Fab'-induced folding of antigenic N-terminal peptides from intrinsically unstructured HIV-1 Tat protein revealed by X-ray crystallography J Mol Biol 2011 405 33 42 10.1016/j.jmb.2010.10.033 21035463

[B57] Serrière J Fenel D Schoehn G Gouet P Guillon C Biophysical characterization of the Feline Immunodeficiency Virus p24 Capsid protein conformation and in vitro capsid assembly PLoS One 2013 8 e56424 10.1371/journal.pone.0056424 23457565 PMC3574121

[B58] Kabsch W Automatic processing of rotation diffraction data from crystals of initially unknown symmetry and cell constants J Appl Crystallogr 1993 26 795 800 10.1107/S0021889893005588

[B59] Keegan RM Winn MD Automated search-model discovery and preparation for structure solution by molecular replacement Acta Crystallogr D Biol Crystallogr 2007 63 447 457 17372348 10.1107/S0907444907002661

[B60] Morris RJ Perrakis A Lamzin VS ARP/wARP and automatic interpretation of protein electron density maps Methods Enzymol 2003 374 229 244 14696376 10.1016/S0076-6879(03)74011-7

[B61] Adams PD Afonine PV Bunkoczi G Chen VB Davis IW Echols N Headd JJ Hung LW Kapral GJ Grosse-Kunstleve RW PHENIX: a comprehensive Python-based system for macromolecular structure solution Acta Crystallogr D Biol Crystallogr 2010 66 213 221 10.1107/S0907444909052925 20124702 PMC2815670

[B62] Emsley P Cowtan K Coot: model-building tools for molecular graphics Acta Crystallogr D Biol Crystallogr 2004 60 2126 2132 10.1107/S0907444904019158 15572765

[B63] Nicholls A Sharp KA Honig B Protein folding and association: insights from the interfacial and thermodynamic properties of hydrocarbons Proteins 1991 11 281 296 10.1002/prot.340110407 1758883

[B64] Morris GM Goodsell DS Halliday RS Huey R Hart WE Belew RK Olson AJ Automated docking using a Lamarckian genetic algorithm and empirical binding free energy function J Computational Chemistry 1998 19 1639 1662 10.1002/(SICI)1096-987X(19981115)19:14<1639::AID-JCC10>3.0.CO;2-B

[B65] Morris GM Huey R Lindstrom W Sanner MF Belew RK Goodsell DS Olson AJ Autodock4 and AutoDockTools4: automated docking with selective receptor flexibility J Computational Chemistry 2009 30 2785 2791 10.1002/jcc.21256 PMC2760638 19399780

[B66] Gouet P Courcelle E ENDscript: a workflow to display sequence and structure information Bioinformatics 2002 18 767 768 10.1093/bioinformatics/18.5.767 12050076

[B67] ESPripthttp://espript.ibcp.fr

[B68] Protein Data Bankhttp://www.rcsb.org

