# Supplementary material for: Biophysical characterization and crystal structure of the Feline Immunodeficiency Virus p15 matrix protein
Source: Retrovirology. 2013 Jun 24;10:64. doi: 10.1186/1742-4690-10-64 (PMC3706335; doi:10.1186/1742-4690-10-64)
Supplement: Additional file 1: Figure S1 — (A) Cross-linking experiment on p15-Δ120 in MES buffer pH 6 at 3 mg/ml (lane 1 & 4) or 6 mg/ml (lane 2 & 5) in the absence (lanes 1–2) or the presence (lanes 4–5) of cross-linking agent BS3. Lane 3: molecular weight marker. (B) Chemical cross-linking with BS3 of control protein FIV p24 [57] in its own buffer (lane 1) or crystallization condition of p15 (0.2 M sodium acetate pH 4.6, 20% w:v PEG 3350, lane 2). Lane 3: molecular weight marker. The asterisk indicates the expected size for the dimeric forms for each protein. Figure S2: Comparison of the dimeric interface of EIAV (A) with the interface 3 of full-length p15 (B) and p15-Δ120 (C). The color scheme is identical to Figure 2A. [file 1742-4690-10-64-S1.doc]

A 26 kDa

*


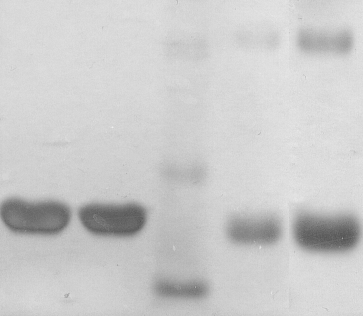


17 kDa

10 kDa

1 2 3 4 5

**B**

**1 2 3**

72kDa

**
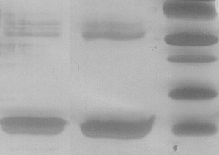
**

*

55kDa

45kDa

34kDa

26kDa

**Supplementary Figure 1**: (A) Cross-linking experiment on p15-Δ120 in MES buffer pH 6 at 3 mg/ml (lane 1 & 4) or 6 mg/ml (lane 2 & 5) in the absence (lanes 1-2) or the presence (lanes 4-5) of cross-linking agent BS^3^. Lane 3: molecular weight marker. (B) Chemical cross-linking with BS^3^ of control protein FIV p24 [57] in its own buffer (lane 1) or crystallization condition of p15 (0.2M sodium acetate pH 4.6, 20% w:v PEG 3350, lane 2). Lane 3: molecular weight marker. The asterisk indicates the expected size for the dimeric forms for each protein.


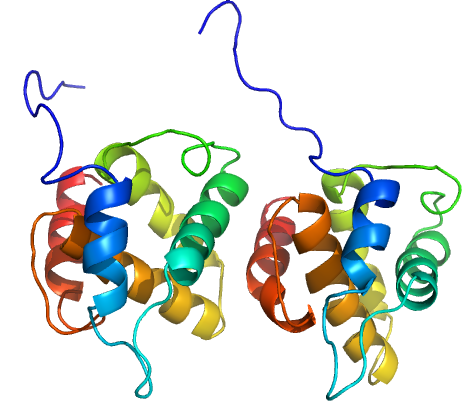
A


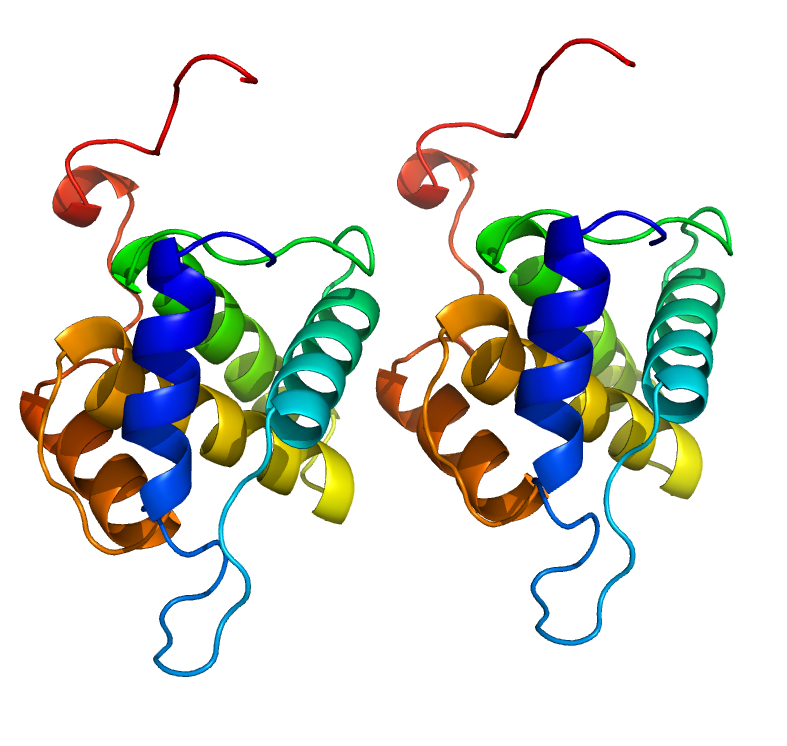


B

C


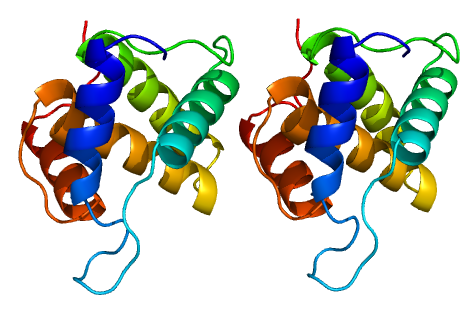


**Supplementary Figure 2:** Comparison of the dimeric interface of EIAV (A) with the interface 3 of full-length p15 (B) and p15-Δ120 (C). The color scheme is identical to Fig. 2A.
